# Weak correlations between serum and cerebrospinal fluid levels of estradiol, progesterone and testosterone in males

**DOI:** 10.1186/s12868-019-0535-3

**Published:** 2019-10-16

**Authors:** Jan Martin, Eva Plank, Bettina Jungwirth, Alexander Hapfelmeier, Armin Podtschaske, Simone M. Kagerbauer

**Affiliations:** 1 Department of Anesthesiology and Intensive Care Medicine, Technical University of Munich, School of Medicine, Klinikum rechts der Isar, Ismaninger Strasse 22, 81675 Munich, Germany; 2 Institute of Medical Informatics, Statistics und Epidemiology, Technical University of Munich, School of Medicine, Klinikum rechts der Isar, Ismaninger Str. 22, 81675 Munich, Germany

**Keywords:** Steroids, Estradiol, Progesterone, Testosterone, Cerebrospinal fluid, Blood, Correlation, Human

## Abstract

**Background:**

Neuroactive steroids seem to be implicated in a variety of neurophysiological and behavioral processes, such as sleep, learning, memory, stress, feeding and aging. Numerous studies have also addressed this implication in various cerebral disorders and diseases. Yet, the correlation and association between steroids in the periphery, e.g. blood, and the central compartments, e.g. cerebrospinal fluid (CSF), have not yet been comprehensively assessed. As the brain is not directly accessible, and the collection of human CSF usually requires invasive procedures, easier accessible compartments, such as blood, have always attracted attention. However, studies in humans are scarce. In the present study we determined estradiol, progesterone and testosterone levels in CSF and serum of 22 males without cerebral disorders or diseases.

**Results:**

Samples were taken under conditions corresponding closest to basal conditions with patients expecting only spinal anesthesia and minor surgery. All samples per patient were collected concomitantly. Total estradiol, progesterone and testosterone concentrations were measured by electro-chemiluminescence immunoassay. The strength of correlation was assessed by Spearman’s rank correlation coefficient. Correlation analysis revealed merely weak to very weak correlations for estradiol, progesterone and testosterone respectively between the CSF and serum compartments.

**Conclusions:**

Total steroid levels of estradiol, progesterone and testosterone in CSF and serum of males without neurological disorders were determined. Weak to very weak correlations between CSF and serum were found thus suggesting that concentrations in the periphery do not parallel concentrations in the central compartments. Further research is needed to clarify to what extent and under which conditions serum levels of estradiol, progesterone and testosterone may possibly serve as a biomarker reflecting the respective concentrations in the CSF or in the brain.

## Background

Neuroactive steroids seem to be implicated in a variety of neurophysiological and behavioral processes, such as sleep, learning, memory, stress, anxiety, feeding, aging [1–3]. Numerous studies have addressed this implication in various disorders and diseases. In psychiatric disorders, e.g. posttraumatic stress disorder [4], affective disorders [5] or suicidality [6], alterations of steroid levels in the cerebrospinal fluid (CSF) were detected. In postmenopausal women with Alzheimer’s disease a clinical study by Schonknecht et al. [7] indicated an association between 17beta-estradiol concentrations in CSF and hippocampal glucose metabolism. In relapsing–remitting multiple sclerosis a clinical study in male patients showed that 17beta-estradiol CSF concentrations are affected [8]. In rodents with experimental subarachnoid hemorrhage Lin et al. [9–11] investigated the beneficial effect of 17-beta-estradiol in attenuating cerebral vasospasm.

However, a general dilemma of human studies on neuroactive steroids is that the brain is not directly accessible. Even presuming that steroid levels in the CSF may reflect steroid activity in the brain, the dilemma in principle persists as human CSF also is not easy to obtain and usually requires invasive procedures such as lumbar puncture or ventricular drainage. Thus, the issue of whether concentrations of steroids in easier accessible compartments, such as blood, may adequately reflect neuropeptide activity in the CSF remains subjected to studies [12].

The present study determined levels of estradiol, progesterone and testosterone in CSF and blood of males without neurological disorders or diseases. The aim of the study was to evaluate the correlations of these levels between the two compartments.

## Methods

The study was approved by the ethics committee of the medical faculty of the Technische Universität München (Project number 2410/09). CSF and blood samples of 22 male patients (mean age 59 years, range 20 to 88 years) were prospectively analyzed. Written informed consent was provided by all patients. CSF and blood samples were obtained concomitantly during spinal anesthesia for elective minor urologic or orthopedic surgery. All patients were otherwise healthy. None of the patients suffered from neurological or psychiatric disorders. CSF samples were taken before the intrathecal injection of the local anesthetic. All samples were collected into plastic serum tubes and analyzed immediately.

Total estradiol, progesterone and testosterone concentrations in CSF and serum were measured on a Roche Mannheim Cobas e 411 immunoassay analyzer using an electro-chemiluminescence immunoassay (detection limit of 5 pg/ml for estradiol, detection limit of 0.05 ng/ml for progesterone, detection limit of 0.025 ng/ml for testosterone).

Strength of bivariate monotonous correlation of quantitative data was assessed by Spearman’s rank correlation coefficient. An absolute value of the correlation coefficient of 0.00–0.19 indicates a very weak correlation, 0.20–0.39 a weak correlation, 0.40–0.59 a moderate correlation, 0.60–0.79 a strong correlation and a value of 0.80–1.0 a very strong correlation. Exploratory hypothesis testing was performed on two-sided 5% significance levels and R version 3.5.0 (R Foundation for Statistical Computing, Austria, Vienna) was used for all computations.

## Results

Patient characteristics are listed in Table 1. The descriptive statistics of the study group are described in Table 2. Spearman’s rank correlation coefficients between serum und CSF concentrations of estradiol, progesterone and testosterone are presented in Table 3. The correlation analysis of estradiol, progesterone and testosterone levels in serum and CSF revealed only weak to very weak correlations between the two compartments with coefficients below 0.3 for all three steroids.

**Table 1:** Patient data

	Age	Operation	Estradiol		Progesterone		Testosterone	
			in serum	in CSF	in serum	in CSF	in serum	in CSF
1	62	TUR prostate	19.9	< 5.0	0.8	< 0.03	4.4	0.3
2	87	TUR bladder	39.6	< 5.0	0.3	< 0.03	6	0.2
3	79	TUR bladder	44.4	< 5.0	0.2	< 0.03	3.9	0.2
4	78	TUR bladder	43.7	< 5.0	0.6	< 0.03	14	0.4
5	79	TUR prostate	6.4	< 5.0	0.1	< 0.03	1	0.14
6	81	TUR bladder	23.5	< 5.0	0.2	<0.03	4.4	0.2
7	55	Metal removal leg	24.8	< 5.0	0.7	< 0.03	4	0.2
8	49	Testicle surgery	27.6	< 5.0	0.84	< 0.03	6.1	0.2
9	20	Knee arthroscopy	26.8	< 5.0	1.1	0.031	3.1	0.2
10	39	Knee arthroscopy	25.6	9.5	0.7	< 0.03	2.4	0.2
11	55	Osteosynthesis leg	31.7	6.4	0.3	< 0.03	6.8	0.22
12	83	TUR prostate	21.7	13.6	0.2	< 0.03	0.2	0.30
13	21	Osteosynthesis leg	36.9	< 5.0	0.8	0.03	6.5	0.2
14	88	TUR bladder	54.3	5.4	0.3	< 0.03	6.8	0.16
15	58	Knee arthroscopy	24.6	< 5.0	0.1	< 0.03	3.3	0.1
16	63	Knee prothesis	53.5	< 5.0	0.7	< 0.03	6.8	0.2
17	68	TUR prostate	17.5	< 5.0	0.5	0.08	4.3	0.2
18	45	TUR bladder	22.3	< 5.0	0.6	< 0.03	4.9	0.2
19	72	TUR bladder	24.8	< 5.0	0.5	< 0.03	4.2	0.1
20	65	TUR bladder	25.8	< 5.0	0.6	< 0.03	4.5	0.2
21	23	Metal removal leg	23	< 5.0	1	< 0.03	4.3	0.2
22	41	Metal removal leg	16.9	< 5.0	0.5	< 0.03	2.1	0.2

*TUR* transurethral resection

**Table 2:** Descriptive statistics

	Median	Mean	Minimum	Maximum	Interquartile range^a^	
Estradiol in serum (pg/ml)	25.2	28.9	6.4	54.3	22.3	36.9
Progesterone serum (ng/ml)	0.6	0.5	0.1	1.1	0.3	0.7
Testosterone in serum (ng/ml)	4.4	4.7	0.2	14.0	3.3	6.1
Estradiol in CSF (pg/ml)	< 5.0	5.7	< 5.0	13.6	< 5.0	< 5.0
Progesterone in CSF (ng/ml)	< 0.03	< 0.03	< 0.03	0.08	< 0.03	< 0.03
Testosterone in CSF (ng/ml)	0.2	0.2	0.1	0.4	0.2	0.2

Descriptive statistics of the collective (n = 22)

*CSF* cerebrospinal fluid

^a^Deviation between the 25th and the 75th percentiles

**Table 3:** Correlation analysis

Estradiol (serum)	Estradiol (CSF)	rho = 0.120	p = 0.595
Progesterone (serum)	Progesterone (CSF)	rho = 0.212	p = 0.344
Testosterone (serum)	Testosterone (CSF)	rho = 0.236	p = 0.290

Spearman rank correlation analysis of CSF and serum levels revealed no strong correlation for estradiol, progesterone or testosterone between serum and CSF. An absolute value of the correlation coefficient (rho) of 0.00–0.19 indicates a very weak correlation, 0.20–0.39 a weak correlation, 0.40–0.59 a moderate correlation, 0.60–0.79 a strong correlation and a value of 0.80–1.0 a very strong correlation

*CSF* cerebrospinal fluid

## Discussion

In humans, the correlation and association between steroids in the periphery, e.g. blood, and the central compartments, e.g. CSF, have not yet been comprehensively assessed. This issue has long been addressed and remains subjected to studies in order to shed light on a complex system of peripheral and central steroid synthesis and steroid penetration across the blood–brain and blood–CSF barrier [12, 13]. This investigation analyzed the correlation of estradiol, progesterone and testosterone levels between CSF and serum of males without cerebral disorders or diseases. The results showed weak to very weak correlations between the two compartments.

An early study in humans by Backstrom et al. [13], published in 1976, found a clear relationship between levels of estradiol, progesterone and testosterone in CSF and plasma unbound and total concentrations. The findings by Backstrom et al. differ from the results of this study. These differences may be attributable to a differing gender distribution in the study groups as Backstrom et al. included predominantly females. In the present study, only males were investigated. It should also be noted that in the study by Backstrom et al. samples from patients with headache investigations or patients with neuroses were analyzed [13] while in the present study only patients without neurological diseases were included. In fertile and postmenopausal females, Molnar et al. [14] determined steroid levels in CSF and blood by radioimmunoassay and reported modest correlations for estradiol and progesterone between CSF and serum while the correlation for testosterone was weak. Again, the patients included in the study by Molnar et al. [14] suffered from a variety of neurological disorders and diseases, predominantly headaches but also epilepsy, brain tumors or cerebral atrophy. In this discussion on correlations and associations between steroids levels in peripheral and central compartments Kancheva et al. [12, 15] argue that some peripheral steroids penetrate the blood–brain barrier providing at least substances for the central nervous system metabolome and thus the predictive value of peripheral steroids appears to be comparable with that of the CSF steroids. Kancheva et al. investigated levels of unconjugated steroids between CSF and serum in males and postmenopausal females undergoing ventriculostomy due to obstructive hydrocephalus [12]. For these measurements, Kancheva et al. used gas-chromatography–mass spectrometry and radioimmunoassay showing several strong correlations between CSF and serum free steroids although demonstrating a very weak correlation for progesterone and a weak correlation for testosterone [12]. In male adults affected by relapsing–remitting multiple sclerosis Caruso et al. [8] investigated neuroactive steroids by liquid chromatography–tandem mass spectrometry in CSF and plasma and observed that the changes in CSF were only partially reproduced in plasma. In another study by Caruso et al. [16], however in rodents, neuroactive steroid levels in plasma and CSF did not fully correlate with their levels in central nervous system and peripheral nervous system tissues. Caruso et al. also analyzed neuroactive steroid levels in plasma and CSF of patients treated with finasteride for androgenic alopecia and report that, even after discontinuation of the drug, progesterone and dihydrotestosterone are increased and decreased respectively in CSF but unaffected in plasma while 17β-estradiol levels were increased in plasma but unchanged in CSF [17]. In an investigation in women with functional hypothalamic amenorrhea Brundu et al. [18] demonstrated a stronger increase of cortisol concentrations in CSF than in serum compared to the respective cortisol levels in eumenorrheic women. Although, in the view of Brundu et al. the mechanisms sustaining this gradient between the peripheral circulation and the CSF were beyond the scope of their investigation, this data illuminate the potential discordance between central and peripheral compartments [18]. Interestingly, in a study on patients with traumatic brain injury, Santarsieri et al. showed a strong correlation between CSF and serum progesterone levels in patients with brain injury while there was barely a modest correlation in healthy controls [19]. Similarly, a modest correlation for cortisol was seen in the patients with brain injury while the correlation was very weak in healthy controls [19].

In view of the numerous studies discussing the alteration of steroid levels in the CSF of patients with neurological disorders [4–6, 20, 21], the comparability across investigations in patients with and without neurological disorders appears limited. These considerations apply and complicate the interpretation of the results of the present study as patients in this cohort did not suffer from neurological or psychiatric diseases, and CSF was obtained in the context of spinal anesthesia and not for diagnostic purposes. Unfortunately, investigations in healthy humans on steroid levels in CSF and blood are scarce.

The discussion and careful interpretation of the results of the present study must also take into account general considerations on the concepts on steroid metabolism in central and peripheral compartments. In the periphery, the steroid hormones progesterone, testosterone and estradiol are synthesized from cholesterol in classic steroidogenic tissues, e.g. in the adrenal, gonad and placenta, and may exert their effects in the periphery. Peripheral steroid hormones may cross the blood–brain barrier and act centrally on brain tissues to regulate neuronal functions [22, 23]. It is also understood and widely agreed that steroids reaching the brain from the periphery may be further metabolized in the brain [24–26]. Steroids can also be synthesized within the brain de novo [2, 26–28], among them pregnenolone, dehydroepiandrosterone, progesterone, testosterone and 17β-estradiol [29, 30]. This steroidogenesis proceeds in neurons and glial cells although the mechanisms controlling and regulating this synthesis remain mostly unclear [23–26, 31]. In addition, a conversion of steroids, e.g. dehydroepiandrosterone, testosterone and progesterone by neural cells into active metabolites, also occurs [29, 32]. Thus, synthesis and conversion of steroids into metabolites further increases the complexity in which these molecules affect the function of the nervous system. It is further discussed by Giatti et al. [33] whether the central synthesis of steroids may be affected by steroids in the periphery resulting in central steroid concentrations that do not parallel the concentrations in the periphery. In the above mentioned study by Kancheva et al. [12] rather strong correlations were found for the α/β-hydroxy metabolites of dehydroepiandrosterone suggesting a relatively uncomplicated transport between CSF and the peripheral circulation. In the view of Kancheva et al. [12], borderline but relatively strong correlations of pregnenolone and dehydroepiandrosterone serum conjugates, being primarily of adrenal origin, with the free steroids in the CSF may reflect the differences in the activity of the adrenal cortex between the subjects. Caruso et al. demonstrated in male adults with relapsing–remitting multiple sclerosis that while an increase of pregnenolone and decrease of dihydroprogesterone and tetrahydroprogesterone occurred in both plasma and CSF, progesterone levels were not modified in CSF, and levels of dihydroprogesterone were significantly increased in plasma but decreased in CSF [8]. In an investigation in females with polycystic ovary syndrome, Kawwass et al. [34] discuss increased CSF levels of estradiol and testosterone and draw attention to the complex effects that testosterone may have on the hypothalamic cascade.

The present study, with respect to the steroids estradiol, progesterone and testosterone, demonstrated merely weak to very weak correlations between serum and cerebrospinal fluid steroid levels. The careful comparison of the results of the present study with the results of the above mentioned studies [12–14, 19] that demonstrated differing strengths of correlation for various steroids underline the caution required when discussing whether steroids concentrations in the periphery may reflect concentrations in the CSF or in the brain. As peripheral and central metabolism or synthesis of steroids such as estradiol, progesterone and testosterone may occur concurrently, serum concentrations may not adequately reflect levels in the CSF unless a rapid restoration of equilibrium between the respective compartments takes place. In addition, there is uncertainty about the extent to which steroid concentrations in the CSF and the brain correlate [33]. In this context, the differentiation between penetration of the blood–brain barrier and penetration of the blood–CSF barrier requires research. Another issue is a better understanding of membrane transport across the blood–brain-barrier [35]. Furthermore, Giatti et al. [33] point out that the varying metabolic pathways, possibly differing depending on brain area or gender, illustrate the intricacy of steroid metabolism.

The strength of the present study is the study cohort consisting of males without neurological disorders since, to our knowledge, investigations on the correlations of steroids levels in concomitantly collected samples of CSF and blood in healthy humans are very scarce. A further strength of the study is that samples were taken under conditions corresponding closest to basal conditions with patients expecting only spinal anesthesia and minor surgery. In contrast, in the above mentioned studies patients usually suffered from neurological disorders or diseases; thus, in such studies, a possible independent regulatory capacity of steroid metabolism patterns in central and peripheral compartments may have been affected by disease-related patterns.

Still, limitations of this study must be considered. The validity of steroid analysis has long been a matter of debate and the literature reveals the use of various detection methods [12], e.g. radioimmunoassay, enzyme immunoassay, liquid chromatography, gas chromatography–mass spectrometry, competitive protein binding assay. The electro-chemiluminescence immunoassay used in the present study was also used by Schonknecht et al. [7] for the measurement of estradiol levels in CSF of humans with Alzheimer`s disease. An enzyme immunoassay for the measurements of estradiol and testosterone as well as cortisol concentrations in CSF were used by Kawass et al. and Brundu et al. respectively [18, 34]. Yet, we acknowledge that methodological deficiencies cannot be excluded. Furthermore, in this study, total steroid levels were measured. In a study on cortisol levels in blood of brain-injured patients, Savaridas et al. [36] demonstrated an increased concentration of the free fraction of cortisol while total serum cortisol was not elevated. Although Savaridas et al. [36] calculated the free fraction of cortisol from total cortisol and corticosteroid-binding globulin it should be noted that measuring total steroid concentrations might not detect changes in the free fraction. But again, in the study by Savaridas et al., brain-injured patients were studied and it remains questionable whether these results can be transferred to patients without neurological disorders. Lastly, with respect to the known issue that the human brain is usually not directly accessible, we reiterate that concentrations of estradiol, progesterone and testosterone were measured in the CSF obtained by lumbar puncture and are thus coupled with the uncertainty that these levels may adequately reflect the respective brain steroid concentrations. Thus, the findings of this study are confined to the correlations between CSF and serum. However, some studies discuss that CSF measurements might be a surrogate for estimating free drug concentrations in the brain [37–39].

## Conclusions

Total steroid levels of estradiol, progesterone and testosterone in CSF and serum of males without neurological disorders were determined. Samples were taken under conditions corresponding closest to basal conditions. Merely weak to very weak correlations between CSF and serum were found thus suggesting that concentrations in the periphery do not parallel concentrations in the central compartments. Further research is needed to clarify to what extent and under which conditions serum levels of estradiol, progesterone and testosterone may possibly serve as a biomarker reflecting the respective concentrations in the CSF or in the brain.

## Data Availability

The datasets supporting the conclusions of this article are included within the article. The datasets used and/or analyzed during the current study are available from the corresponding author on reasonable request.

## Abbreviations

CSF: cerebrospinal fluid
TUR: transurethral resection
